# Integrated fiber optical receiver reducing the gap to the quantum limit

**DOI:** 10.1038/s41598-017-02870-2

**Published:** 2017-06-01

**Authors:** Horst Zimmermann, Bernhard Steindl, Michael Hofbauer, Reinhard Enne

**Affiliations:** TU Wien, Institute of Electrodynamics, Microwave and Circuit Engineering, Gusshausstraße 25/354, 1040 Vienna, Austria

## Abstract

Experimental results of a single-photon avalanche diode (SPAD) based optical fiber receiver integrated in 0.35 µm PIN-photodiode CMOS technology are presented. To cope with the parasitic effects of SPADs an array of four receivers is implemented. The SPADs consist of a multiplication zone and a separate thick absorption zone to achieve a high photon detection probability (PDP). In addition cascoded quenchers allow to use a quenching voltage of twice the usual supply voltage, i.e. 6.6 V instead of 3.3 V, in order to increase the PDP further. Measurements result in sensitivities of −55.7 dBm at a data rate of 50 Mbit/s and −51.6 dBm at 100 Mbit/s for a wavelength of 635 nm and a bit-error ratio of 2 × 10^−3^, which is sufficient to perform error correction. These sensitivities are better than those of linear-mode APD receivers integrated in the same CMOS technology. These results are a major advance towards direct detection optical receivers working close to the quantum limit.

## Introduction

Optical receivers implementing PIN and linear-mode avalanche photodiodes (APDs) are very mature even as optoelectronic integrated circuits (OEICs). Due to electronic noise in amplifier circuits and due to excess noise of APDs there is however still a rather wide gap to the quantum limit set by photon statistics. From this Poisson statistics follows that a mean value of 21 photons is necessary in a logical-“1” bit to allow for a bit-error ratio (BER) of 10^−9^ according to ref. 1 (see also Supplementary Section I). An analog receiver with an integrated avalanche photodiode having a diameter of 200 µm in 0.35 µm CMOS achieved a sensitivity of −31.8 dBm at 1 Gbit/s for a wavelength of 675 nm and for BER = 10^−9^ ^2^. This sensitivity value corresponds to an average optical power of 0.66 µW, from which we can calculate (number of photons n_ph_ = 2 < P > t_bit_/E_ph_; < P > is average optical power, t_bit_ = 1 ns for 1 Gbit/s in non-return to zero; E_Ph_ is photon energy; the factor 2 is necessary because the optical power in a logical-“1” bit is twice the average optical power) that 4,500 photons are necessary to receive one logical-“1” bit with BER = 10^−9^. Furthermore, the trend in pure micro- and nanoelectronics since many years is from analog to digital signal processing. Nowadays, single-photon avalanche photodiodes (SPADs) operated at higher reverse bias (above the breakdown voltage V_bd_ of APD) in the Geiger mode realize a much larger gain than APDs^3^ and relax the requirements of analog amplifiers or even avoid them completely. The SPAD practically delivers a digital signal when a photon is absorbed and triggers a self-sustaining avalanche. A quenching circuit is needed to stop the self-sustaining avalanche by reducing the reverse bias voltage to below V_bd_^4, 5^. Then the SPAD is charged again to V_plus_ with increasing its reverse bias by the quenching voltage to make it sensitive again. Therefore, SPAD-based optical receivers are neither limited by thermal and shot noise nor by APD excess noise and it was assumed that they can save electrical power and chip area compared to conventional analog amplifiers. The avoidance of APD excess noise, the idea of saving chip area and electrical power as well as the low fabrication costs of CMOS circuits (for creation of mass markets) and the idea to improve the sensitivity of CMOS receivers over that of APD CMOS receivers were the main motivation for this work.

However, there are parasitic effects in SPADs: During an SPAD event many charge carriers are generated and these can fill impurity defect states in the semiconductor, from which they are released after some random delay being characterized by a time constant in the order of 8 ns to 1 µs^6–8^. The traps and impurities being responsible for afterpulsing depend on the process used for fabrication of the SPADs and obviously vary strongly from process to process. After being released the charge carriers can trigger new SPAD events, which are called afterpulses. Afterpulsing probabilities from 0.02% to 27% were reported for deadtimes from 6 ns to 500 ns in ref. 9 and references cited therein for SPAD diameters up to 20 µm and excess bias voltages up to 6 V. In ref. 7 the afterpulsing probability was 3.4% reported for a deadtime of 110 ns. For the SPAD with 12 µm diameter investigated in ref. 10, an afterpulsing probability of 0.3% with 300 ns deadtime at 10 V excess bias was found. The afterpulsing probabilities of the SPAD used in the receiver described below in the Results section are well within this range especially when considering the active SPAD area of 3,750 µm².

Moreover, during an avalanche event, photons are emitted^11–13^. When these avalanche-generated photons are absorbed in neighboring SPADs and trigger an SPAD event there, optical cross talk occurs^9^. Optical cross talk in SPAD arrays was found to depend on the wafer thickness^14–16^ and on the excess bias^16^. The optical cross talk was found to increase with decreasing silicon wafer thickness due to total reflection at the silicon surfaces. The optical cross-talk probability between two SPADs having a diameter of 20 µm in a distance of 100 µm was 0.03% for 5 V excess bias for a usual 0.35 µm CMOS wafer^9^. An exponential decay length of 250 µm was reported in ref. 15 for a silicon layer thickness of 10 µm and avalanche-generated photons in the spectral range from 800 to 950 nm were assumed to explain the findings. A measured SPAD emission spectrum even covered the spectral range from 500 to 1100 nm^14^. The measured optical cross-talk probabilities described in the results section below for a usually thick 0.35 µm CMOS wafer are consistent with these findings.

SPAD-based optical receivers were described first in refs 17 and 18. The SPAD receivers used in these references were however limited by p+/(deep)n-well SPADs, which had a thin combined absorption/multiplication zone (see Supplementary Section II for the comparison of thin and thick SPAD), low array fill factor (2.42%, mainly resulting from the main goal to achieve a large dynamic range)^17^, and by non-ideal quenching circuits. At 100 Mbit/s a sensitivity of −31.7 dBm at 450 nm and BER = 10^−9^ was achieved with a 32 × 32 SPAD receiver having a chip area of 2.4 × 2.1 mm^2^ ^17^. Such a sensitivity at 100 Mbit/s, however, easily can be obtained with analog PIN photodiode receivers. A receiver with an array of 100 SPADs was used at 20 Mbit/s with a 860 nm laser^18^, however, no sensitivity was reported. In contrast our main goal was to improve the quencher by using low-voltage transistors of a standard CMOS process and to achieve a high fill factor of the detector array in order to be able to obtain a good sensitivity. Recently, SPADs even were used in receivers exploiting more advanced modulation techniques like 4-PAM (Pulse Amplitude Modulation)^19, 20^ and OFDM (Orthogonal Frequency Division Multiplexing)^21^ in visible light communication via free space. With a 32 × 32 SPAD matrix a receiver in 0.13 µm CMOS^22^ with a chip area of 2.4 × 1.7 mm² achieved a sensitivity of −64 dBm at 100 kbit/s^19^ where the quantum limit is at −95 dBm. A data rate of 200 kbit/s was reported with a 32 × 32 SPAD array having a fill factor of 43%^20^. At a transmission speed of 1 kbit/s a sensitivity of −107 dBm was reported^21^ compared to the quantum limit for 1 kbit/s at −115 dBm.

## Results

### Receiver

The first key idea suggested here in this article is a SPAD with separate thick absorption zone using a 0.35 µm PIN photodiode CMOS process leading to a low capacitance of the SPAD (Supplementary Section II), which in turn reduces the avalanche charge. The second key improvement comes from a quencher with low detection threshold (100 mV) and implementing cascoding to double the quenching voltage V_q_ from the usual 3.3 V supply voltage to 6.6 V, which allows to use excess bias voltages up to almost twice the 3.3 V supply. The low detection threshold helps to reduce the avalanche charge further to obtain a low afterpulsing probability (APP), when the quenching is done faster than the avalanche builds up. The thick absorption region of the SPAD and the doubled quenching voltage lead to a large PDP at 635 nm. The PDP increases rapidly within about 1 V above the breakdown voltage and for further increasing excess bias the slope of the PDP reduces and becomes linear^23^. Because dark count rate (DCR) and APP also increase with the excess bias voltage, a higher excess bias voltage does not necessarily improve the overall SPAD performance^24^. For our SPAD APP dominates over DCR. In ref. 23 the afterpulsing probability of a 0.35 µm high-voltage CMOS SPAD increased slowly in a wide excess bias (V_ex_) range (where it could be approximated by a linear function) and from a certain V_ex_ value on the slope of APP increased rapidly. Consequently, increasing the excess bias voltage does not necessarily improve BER and sensitivity of an SPAD-based receiver. Therefore, we varied the excess bias voltage in steps of 0.5 V by changing the reverse bias voltage of the SPAD to obtain the best BER and sensitivity.

**Figure 1:**
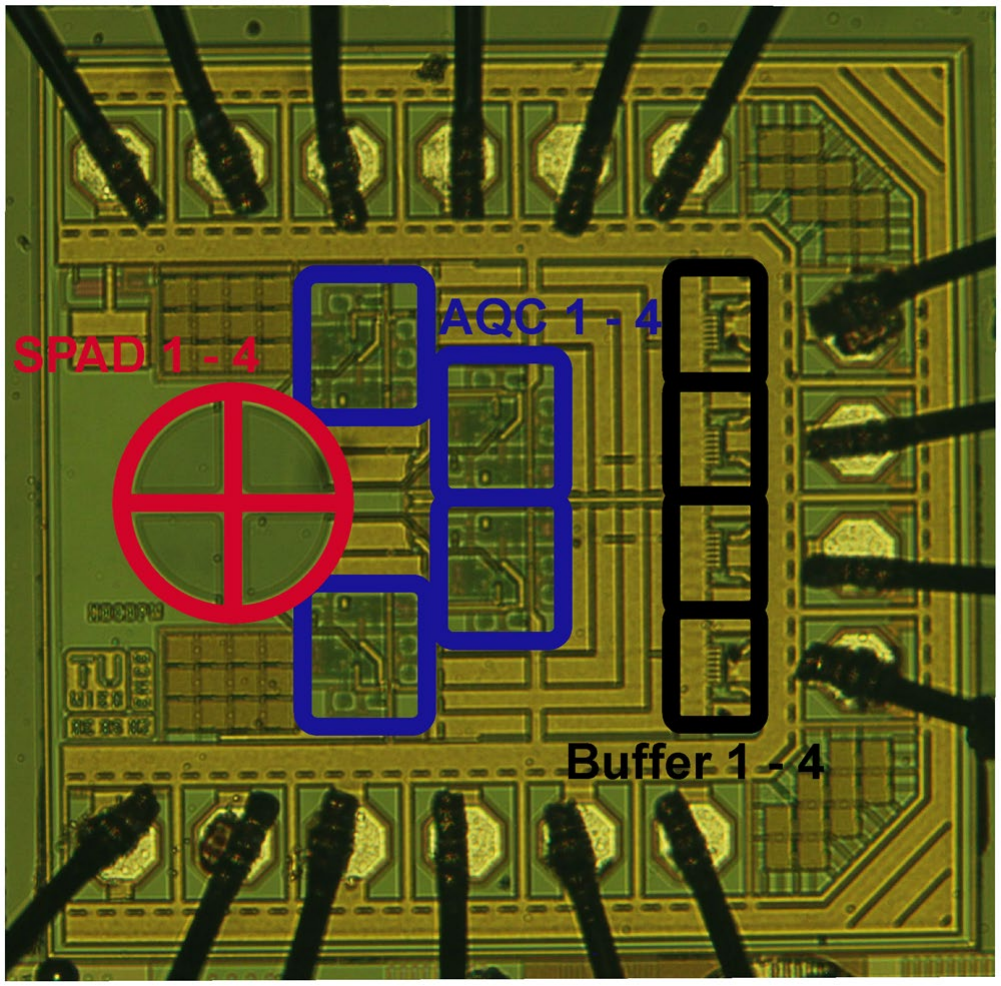
Microphotograph of receiver test chip. The diameter of the 4-SPAD array is 200 µm with a gap of 34 µm between the SPADs. The area of this 4-SPAD array is 31,500 µm² (0.0315 mm²). Each of the four quenchers (AQC) has the dimensions 134 × 130 µm².

Figure 1 shows the chip photo of a 4-channel SPAD-based receiver test chip. The total active area of the SPAD optical receiver is 101,200 µm² or about 0.10 mm² for 4 SPADs and 4 quenchers (without buffers for driving 50 Ω). Inclusive bondpads, 50 Ω output buffers and block capacitors the overall dimensions are 985 × 960 µm² corresponding to 0.95 mm². The APD, TIA and post amplifier (to obtain a digital signal) of an APD OEIC in 0.35 µm CMOS^25^ occupied (without 50 Ω driver) an active area of 120,800 µm² at a total chip area of 1.56 mm². We did not implement a signal processing circuit to combine the four quencher output signals to one output signal hardwired on the receiver test chip in order to have larger flexibility in the choice of the data processing by using Matlab.

**Figure 2:**
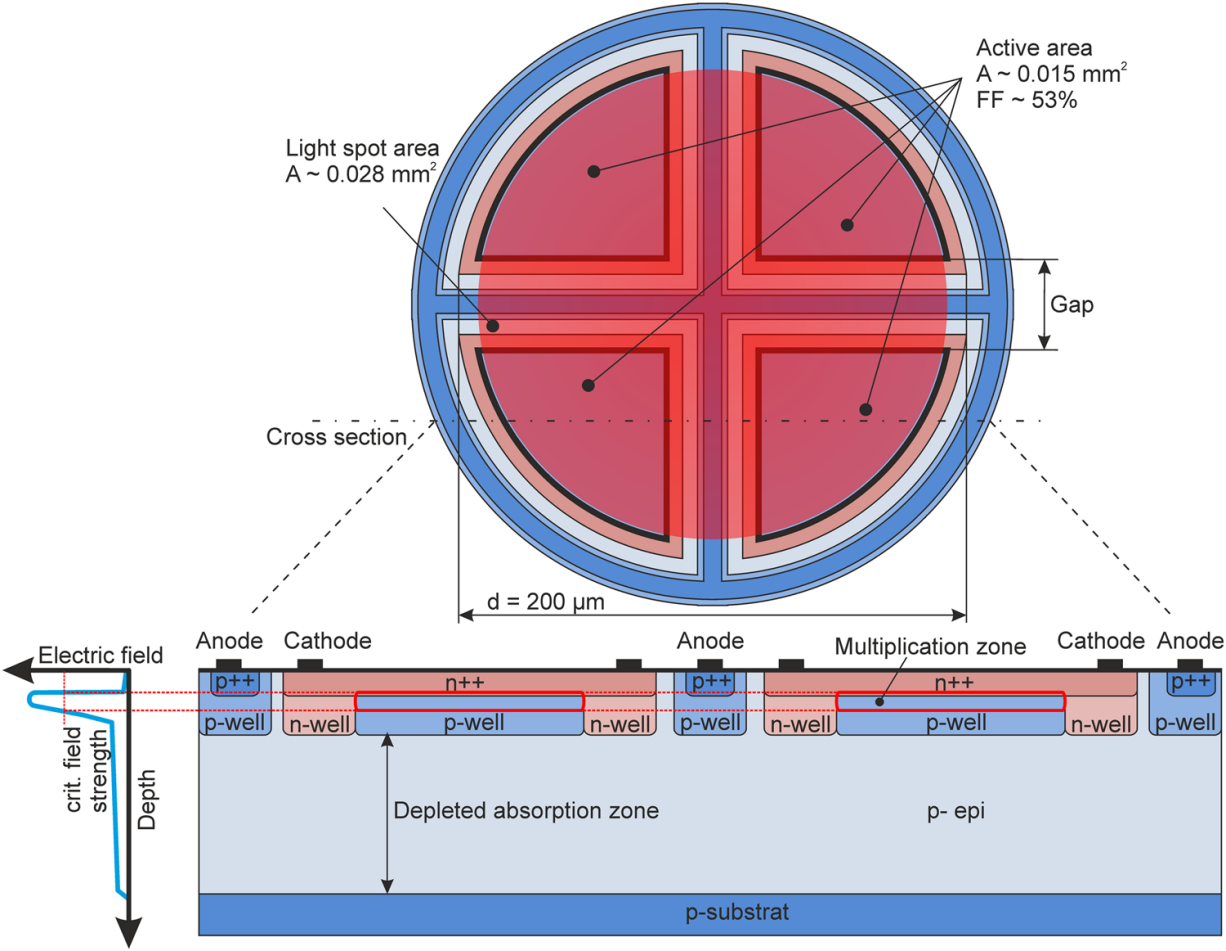
Top view, schematic electric field distribution and cross section of SPAD with separate absorption and multiplication zones (not to scale).

The cross section of the SPAD is shown in Fig. 2. The fill factor (FF) of the 4-SPAD array is 0.53 (see Fig. 2) compared to 1.00 of an APD with the same diameter of 200 µm. The fill factor of the SPAD is about a factor of two smaller and allows to outperform the APD receiver with respect to sensitivity.

The SPAD device (see bottom of Fig. 2) uses the about 12 µm thick low doped (2 × 10^13^ cm^−3^) p- epitaxial layer of a PIN photodiode CMOS ASIC technology as absorption zone. The multiplication zone is located at the n++/p-well junction (see bottom of Fig. 2), where the p-well is doped to the order of 10^17^ cm^−3^. The electrons photogenerated in the thick absorption zone drift upwards into the multiplication zone and can trigger a huge avalanche there. Starting from zero bias voltage the space-charge region extends from the n++/p-well junction with increasing reverse voltage through the p-well and the low doped absorption zone. For the process used, the p-well can be completely depleted already at approximately −16 V and the low doped absorption zone depletes already at a reverse voltage of −18 V. The schematic distribution of the electric field is shown on the left bottom part of Fig. 2. In the linear mode, a bandwidth of 580 MHz was achieved at an avalanche gain of 23^26^. Its breakdown voltage V_bd_ is −25.8 V (Supplementary Section III) and its quantum efficiency for an avalanche gain M = 1 at 635 nm is 74.9%. All SPAD characterizations were done at 25 °C. The PDP of a reference SPAD for 635 nm measured at 0.3 pW ranges from 22.4% at V_q_ = 3.3 V to 36.7% at V_q_ = 6.6 V, its dark count rate (DCR) ranges from 21,500 s^−1^ at V_q_ = 3.3 V to 35,500 s^−1^ at V_q_ = 6.6 V (Supplementary Section V). The APP was determined by measuring photon interarrival times of dark count events^27^. Its APP at a dead time of 9 ns ranges from 0.95% at V_q_ = 3.3 V to 5.1% at V_q_ = 6.6 V, and the optical cross talk probability (CTP) ranges from 0.45% at V_q_ = 3.3 V to 2.3% at V_q_ = 6.6 V for neighboring SPADs in a reference 4-SPAD array (and from 0.14% to 0.60% for diagonal placed SPADs, respectively). The optical cross talk probability was extracted from the dark count rate measurement data. Pulses occurring in two neighboring SPADs at the same time (we counted them when their leading edges were measured within 1 ns) are accounted to be caused by optical cross talk. The number of pulses caused by optical cross talk divided by the total number of pulses defines the optical cross talk probability.

**Figure 3:**
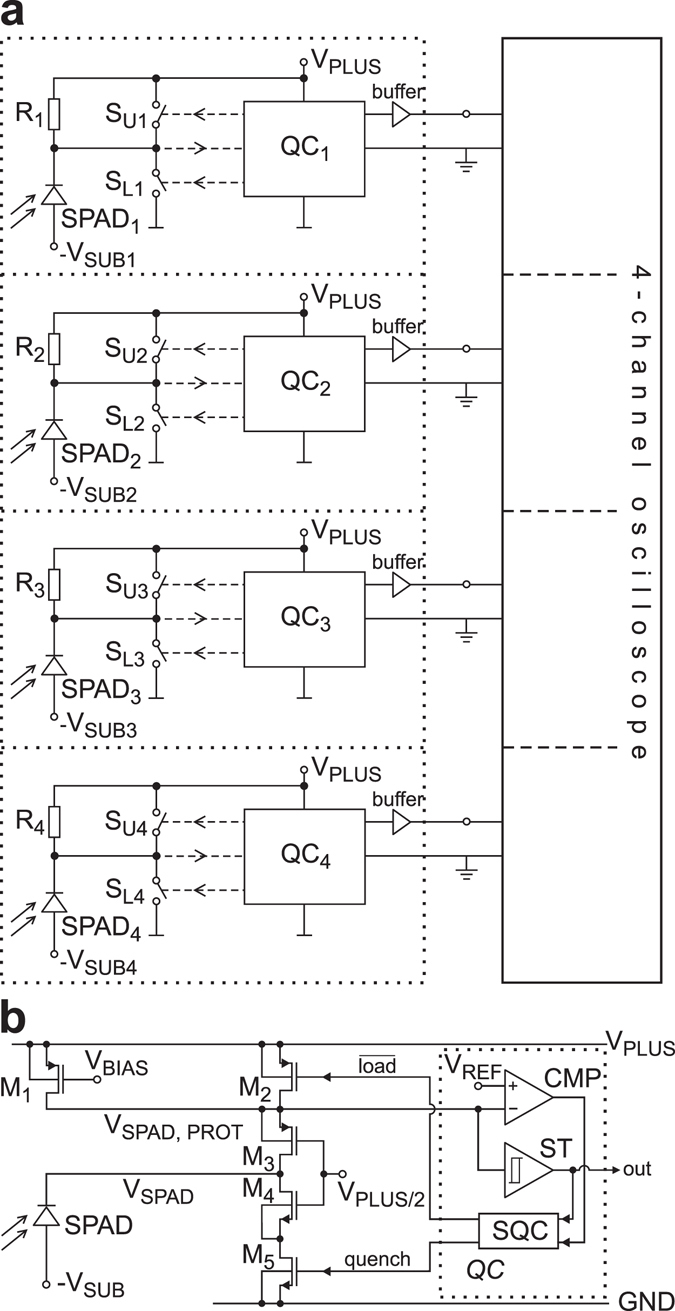
(**a**) Block diagram of 4-channel receiver connected to an oscilloscope, (**b**) quenching circuit.

For a receiver with only one SPAD, an APP of 5.1% results in detection of 51 “1”-“1” when 1000 times “1”-“0” was sent, i.e. the bit-error ratio is 5.1 × 10^−2^ for the second of these two bits. Because of these large APPs and CTPs only one SPAD does not allow a bit-error ratio of 2 × 10^−3^ or lower, as it is required to enable error correction^28, 29^. When designing the test chip, our estimate was that 4 SPADs should be sufficient (see Supplementary Section I), when for a logical “1” each SPAD must detect a photon. Figure 3a depicts the block diagram of the receiver test chip and the implementation details of the SPAD driving and quenching circuitry are depicted in Fig. 3b. Seen from the conceptual domain in Fig. 3a each channel includes an SPAD, a quenching resistor (R), charging (S_U_) and discharging switches (S_L_) and the quencher control QC. In the actual circuitry (Fig. 3b) the charging switch is realized by PMOS transistor M_2_ and the quenching switch by NMOS M_5_. The PMOS M_1_ realizes the quenching resistor. In order to protect the MOSFETs M_1_, M_2_, M_5_ as well as the circuitries of the comparator (CMP) and Schmitt trigger (ST) from the high voltage swings of the SPAD’s cathode the MOSFETs M_3_ and M_4_ are inserted exploiting the technique of cascoding described e. g. in ref. 30. Their gate bias of V_plus_/2 makes them operate as protective cascodes. As a result the lower limit of V_SPAD,prot_ is approximately V_plus_/2 plus the threshold voltage of M_3_ (i.e. the drain-source voltage of the active resistor M_1_ is always smaller than 2.6 V) and the upper limit of the drain voltage of M_5_ is approximately V_plus_/2 minus the threshold voltage of M_4_ (i.e. the drain-source voltage of M_5_ is also always smaller than 2.6 V).

During the three states (i) “charge SPAD”, (ii) “SPAD cathode floating” and (iii) “quenching”, the quenching circuit works like this:

(i) For “charge SPAD”, the load signal is V_plus_/2 and therefore M_2_ is conducting. The source of M_3_ is pulled to V_plus_ and therefore M_3_ is also conducting. The quench signal is at GND and M_5_ is off. Therefore M_4_ is off, since its gate-source voltage is negative. Consequently the cathode of the SPAD is charged to V_plus_.
(ii) For “SPAD cathode floating”, the load signal is at V_plus_ and M_2_ is off. Since M_1_ is operating as an active resistor according to the V_bias_ voltage, the source of M_3_ is far above V_plus_/2 and M_3_ is conducting. Therefore, when the SPAD fires, a current flows through M_3_ and M_1_ and when the potential on the V_SPAD,prot_ line falls below V_ref_ the comparator CMP switches and starts quenching.
(iii) For “quenching”, the quench signal is at V_plus_/2 switching on M_5_. The load signal is at V_plus_ and M_2_ is off. Since M_5_ pulls the source of M_4_ to GND, the gate-source voltage of M_4_ is large (equal to V_plus_/2) and M_4_ is also conducting. Consequently the SPAD is being discharged to below its breakdown voltage and the avalanche current stops.

The quench control block (QC) is built up by a voltage comparator CMP, a fast sequencer SQC which generates the signals for the switches and a Schmitt trigger ST which derives the output signal. According to the mismatch parameters of the process used, the 3σ input DC offset of the comparator is 20 mV (σ is the standard deviation). The whole circuit is optimized that it responds as fast as possible by switching on M_5_ if the SPAD voltage underruns V_ref_. Simulations with layout extracted parasitics and with 60fF SPAD capacitance (Supplementary information, Fig. S2) indicate response times below 560 ps, i.e. the current flow through M_4_ and M_5_ starts 560 ps after the cathode potential fell below V_Plus_ – 100 mV = 6.5 V. The SPAD’s capacitance is discharged to 0 V after further 0.44 ns with a peak current of up to 4.1 mA. Since the comparators input offset is below 20 mV and the SPADs’ voltage swings are faster than 1 V/ns^31–33^, the time variation when the SPAD reaches the comparator’s decision threshold (100+/−20) mV is +/−20 ps. Due to this very fast voltage swing at the comparator’s input, metastability effects can be excluded and the comparator reaches a valid output state quickly.

Consequently, during the first 0.56 ns after absorption of the photon the SPAD quenches passively (whereby M1 first acts as resistor and then limits the current as a current source due to its gate bias voltage to 110 µA when the avalanche current became larger; the avalanche current then discharges the SPAD’s capacitance) and from 0.56 ns to 1.0 ns after absorption of the photon the cascoded quencher takes charges away from the SPAD’s cathode actively through M_4_ and M_5_, i.e. charge being available for the avalanche current through the SPAD is reduced. When considering reported avalanche current rise times of 1.6 ns^31^, 1.28 ns^32^ and 0.7 to 0.8 ns^33^, a large portion of the quenching seems to be passively in our experiments. After M4 and M5 are on, however, the current through M1 is negligible compared to the current through M4 and M5. ref. 34 reports passive and active quenching with active reset. Passive quenching lasted for the first 15 ns after photon absorption and active quenching was finished after additional 2 ns. Compared to ref. 35, where the excess bias is 5 V and active quenching starts after 2.3 ns, the quencher suggested here reduces the quenching time about by a factor of 4.

Each photon detection cycle starts with charging the cathode of the SPAD to V_plus_ (maximum voltage across SPAD V_m_ = V_sub_ + V_plus_) by closing the upper switch (S_U_) for a short period (approximately 1.5 ns). When a photon triggers an avalanche in the SPAD, a signal current starts to flow and causes a voltage drop across the high-ohmic p-channel MOSFET active resistor. In addition the avalanche current discharges the SPAD’s capacitance. The quenching control circuit (QC) detects this event already at 100 mV below V_plus_ and closes the lower switch (S_L_) after 560 ps, which quenches the SPAD by discharging it to below V_bd_. Due to the fast response of the quenching circuit a portion of the charge stored in the diode capacitance goes via the quencher what results in a reduced amount of avalanche carriers in the SPAD. After a defined recovery time of 6.5 ns, the lower switch (S_L_) is opened and the upper switch (S_U_) is closed to charge the SPAD again with a slope of 4.5 V/ns, i.e. within about 1.5 ns. As long as the upper switch is closed its resistance is very small and the avalanche current cannot cause a voltage drop of 100 mV, when a photon triggers the SPAD. Therefore, the comparator cannot detect the event during the period when the upper switch is closed. The resulting total dead time is 9 ns. This value was aimed at to enable a data rate of 100 Mbit/s (requiring a dead time shorter than 10 ns) and on the other hand not to let increase APP strongly for shorter dead times, since APP decays exponentially with time (ref. 8 reported a time constant of 8 ns, requiring a dead time as large as possible). As a consequence, for a bit duration of 20 ns (corresponds to 50 Mbit/s in non-return to zero) and a dead time of 9 ns, in average 45% of the incident photons will be lost during this dead time at low optical power at the sensitivity limit. In return to zero with a duty ratio of 50% or less no photon will be lost at this low optical power. Four identical channels are present on the receiver test chip. The power consumption of each QC obtained by circuit simulation in the idle state (i.e. no SPAD events considered) is 4.78 mW (because of high-speed quenching), leading to a total power consumption of the SPAD receiver of larger than 19.1 mW when SPAD events occur, which corresponds to more than 380 pJ/bit in comparison to 55 pJ/bit of the APD receiver of ref. 25.

### Experiments

**Figure 4:**
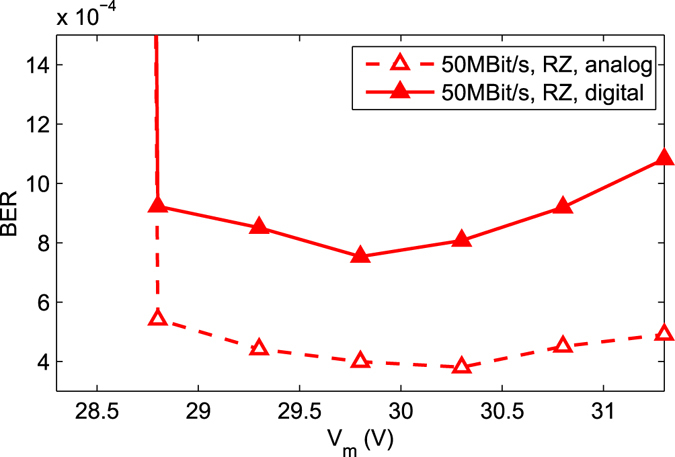
Bit error rate for an optical input power of 7.5 nW.

For all measurements of the receiver the temperature was 25 °C. Four output buffers delivered digital signals to a 4-channel oscilloscope operated at 5 GS/s. The measurement set-up is depicted in Fig. S6 of Supplementary Information. In the experiment the light spot coupled from an optical fiber to the 4-SPAD array within a dark box had a diameter of slightly below 200 µm (see Fig. 2). The fiber was adjusted carefully to ensure equal count rates in the 4 SPADs. A variable attenuator was used and the power at the fiber end was measured with a Thorlabs PM200 optical power meter before adjusting the fiber to the SPAD array. A bit pattern generator modulated the 635 nm light source, consisting of a continuous-wave laser and an external modulator, having an extinction ratio larger than 100, with a 50 Mbit/s non-return to zero (NRZ) stream having a pseudo random bit sequence (PRBS) of the length 2^7^-1. In addition return to zero (RZ) with a duty cycle of 50% was used. At 100 Mbit/s RZ a duty cycle of 10% was used because of the 10 ns duration of a bit and the 9 ns dead time of SPAD and QC. The received bit streams were stored by the oscilloscope and analyzed in MATLAB on a personal computer. The reverse bias voltage of the SPAD was varied in steps of 0.5 V to minimize the BER for constant optical input power. For an example, Fig. 4 shows the BER for 50 Mbit/s with RZ (duty cycle 50%) in dependence on the SPADs’ reverse bias voltage V_m_. For 7.5 nW, the minimum BER occurs at V_m_ = 29.8 V for the digital processing and at V_m_ = 30.3 V for the analog processing. Considering the breakdown voltage of 25.8 V, the excess bias voltages are 4.0 V and 4.5 V, respectively, for the lowest BER with 7.5 nW optical power. It also can be seen from Fig. 4 that the BER does not strongly depend on the excess bias voltage for V_ex_ values between 3.0 V and 5.5 V.

**Figure 5:**
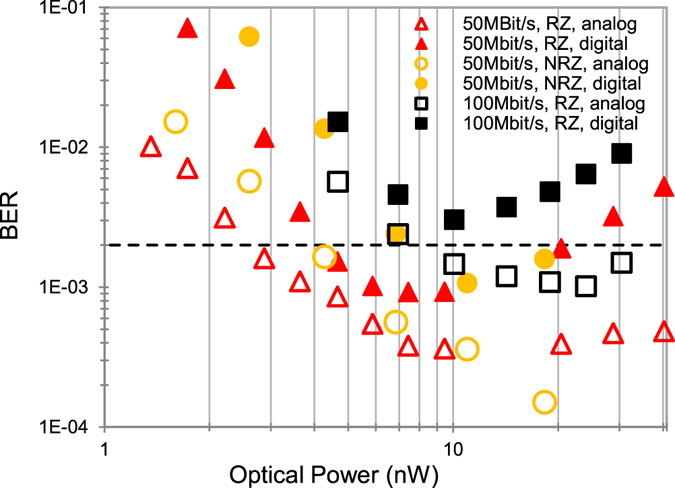
BER versus average optical input power.

Figure 5 shows the BER versus average optical input power. For 50 Mbit/s, BER = 2.0 × 10^−3^ in NRZ at V_m_ = 31.3 V the necessary optical input power is 4.0 nW (−54 dBm) for analog post-processing and 7.6 nW (−51.2 dBm) at V_m_ = 29.8 V with digital processing. In RZ with 50% duty cycle at V_m_ = 30.3 V 2.7 nW (−55.7 dBm), corresponding to a power density of 9.6 µW/cm² which is calculated for the light spot area of 0.028 mm^2^ from Fig. 2) are necessary when analog post-processing is done and in RZ at V_m_ = 30.8 V 4.3 nW (−53.7 dBm) are needed with digital post-processing. For 100 Mbit/s, BER = 2.0 × 10^−3^ with 10% duty cycle at V_m_ = 32.3 V 7.0 nW (−51.6 dBm) are necessary with analog processing and 16.5 nW (−47.8 dBm) with digital processing. An interesting result is that analog post-processing of the quenchers’ output data achieves better sensitivities than digital processing. Another important result is that RZ gives a lower BER than NRZ when comparing BERs at 50 Mb/s (for digital and analog processing). One explanation for this is that more of the incident photons can be detected for a bit duration of 20 ns and a dead time of 9 ns when the photons of a bit are present within a pulse of 10 ns (RZ with duty ratio 50%) instead within a pulse of 20 ns (NRZ). A second reason is given due to the jitter of the SPAD with the thick absorption region (depending on the depth where the photon is absorbed the drift time to the multiplication zone can be up to about 0.3 ns). According to this jitter, the SPAD may fire up to 0.3 ns after the end of the light pulse in NRZ and a logical “1” will be detected in a following “0”-bit, causing a bit error. Such bit errors due to jitter can be avoided by using RZ because there is no light for 10 ns before the “0”-bit starts at 50 Mbit/s with a RZ duty ratio of 50%.

## Discussion

**Figure 6:**
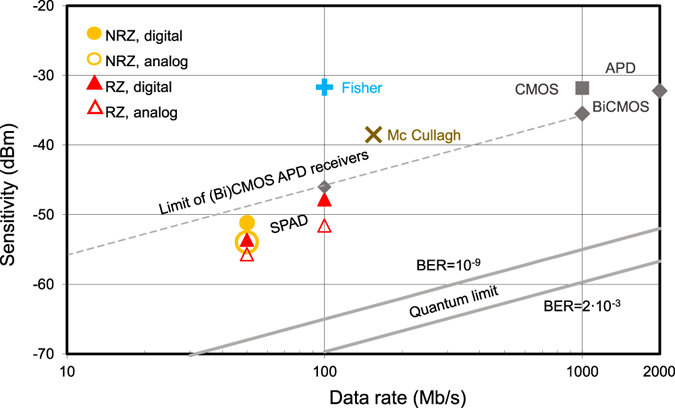
Comparison of sensitivities for receiver OEICs and quantum limit.

Recent publications report on integrated direct detection high-speed receivers with implemented linear-mode APDs, however, with sensitivities of −22 dBm at 2.4 Gbit/s^36^, −4 dBm at 10 Gbit/s^37^ and −7 dBm at 12.5 Gbit/s^38^ due to thin multiplication/absorption zones, i.e. far away from the quantum limit. Coherent receivers can even surpass the quantum limit, however, by using very expensive discrete components^39^. Discrete optical receivers at low data rates using optimal transistors and optimal (with respect to low-field quantum efficiency and excess noise factor) linear-mode APDs were investigated already more than 30 years ago^40–42^. The doping profiles of discrete APDs were optimized for a thicker multiplication zone with a lower maximum electric field strength because then the excess noise of linear-mode APDs is minimized^43^. This leads to a low ratio of the hole-to-electron ionization coefficients k_eff_. This minimization of the APD excess noise and a very thick absorption zone of discrete APDs, however, requires a reverse bias voltage often in excess of 100 V. The reported sensitivities of these discrete receivers are similar (≈−54 dBm) to the ones reported here for the SPAD receiver test chip, but the used discrete APDs with a very thick multiplication zone leading to a ratio of the hole-to-electron ionization coefficients k_eff_ = 0.035 needed a reverse voltage of 350 V^42^. A sensitivity of −35 dBm at 155 Mbit/s was reported with an APD having a diameter of 5 mm^44^. Such optimized discrete APDs implement special doping profiles to form the multiplication zone with optimal electric field and very thick absorption regions which are currently not available in (Bi)CMOS technologies, because of limited breakdown voltages (similar limitations for breakdown hold for integrated linear-mode APDs as explained in section III of Supplememantary Information). Very expensive process modifications would be necessary to implement electric field engineered multiplication regions, to increase the thickness of the isolation stack to prevent opening of parasitic MOS channels between devices by high voltages on metal lines of (Bi)CMOS chips^45^ and lateral distances of devices would have to be increased strongly to avoid reachthrough currents between them^46^ (For an SPAD array this would require a larger gap and a reduced fill factor). Integrated linear-mode APDs cannot use optimized doping regions, because process modifications would be necessary, and they therefore show larger excess noise than discrete APDs. However, due to low fabrication costs of OEICs non-optimum receiver performance nevertheless is interesting for gaining mass markets. Therefore we limit the comparison to receiver OEICs (see Fig. 5). A 0.35 µm APD CMOS receiver achieved a sensitivity of −31.8 dBm at 1 Gbit/s^2^. With the same APD structure and diameter of 200 µm as used for the SPAD receiver here, a receiver OEIC in 0.35 µm BiCMOS achieved sensitivities of −32.2 dBm and −35.5 dBm at 2 Gbit/s and 1 Gbit/s, respectively, for BER = 10^−9^ and 675 nm^26^. k_eff_ for these APD OEICs was between 0.07 and 0.104 ^2^. When estimating the sensitivity of a 0.35 µm CMOS APD receiver at 100 Mbit/s, a sensitivity of −45 dBm to −46 dBm is obtained. The dashed line in Fig. 6 shows the resulting sensitivity limit of CMOS and BiCMOS receivers with integrated linear-mode APDs.

The quantum limit (Supplementary Section I) for 635 nm and BER = 10^−9^ is also shown in Fig. 6 for easy comparison. For 100 Mbit/s and BER = 10^−9^ it is at −65 dBm. When it is calculated for BER = 2 × 10^−3^, a value of −69.7 dBm results for 635 nm and 100 Mbit/s. It can be seen from Fig. 6 that there still remains a gap of more than 10 dB between the quantum limit and the results presented here. However, a large progress compared to ref. 17 is achieved and better sensitivities than with a linear-mode APD in the same CMOS technology are introduced. The chip area is reduced by more than a factor of 4 compared to the 32 × 32 SPAD receivers of refs 17, 19 and 22.

Since the PDP in our experiments is between 22% and 36%, our receiver needs about 5 to 3 times more photons than with an ideal 100% efficiency. Since we have 4 detectors, of which each has to detect a photon, our receiver needs another factor of 4 more photons than indicated by the quantum limit. Due to PDP and 4 detectors, a sensitivity being a factor in the range from 12 to 20 above the quantum limit would be expected. Due to dead time and jitter an even larger gap of a factor of about 50 (17 dB) results. The PDP and low capacitance due to the thick absorption zone of the SPAD as well as the increase of the excess bias voltage due to the cascoded quencher contribute more to the reduction of the gap to the quantum limit than the speed of the quenching circuit. Considering that SPADs with better PDP, DCR and APP were described in the literature, there is hope that binary SPAD-based integrated optical receivers can come even closer to the quantum limit than it is reported here. The results indicate that a better analog processing method of the quenchers’ output data is desirable. It may even be possible to surpass the quantum limit by using OFDM, which increases the channel capacity. However, as a conclusion of the above reported quenchers’ power consumption being necessary for fast quenching it is difficult to design digital SPAD receivers with lower power consumption than conventional analog receivers. The used functionality of oscilloscope and Matlab computing (both only present in this first investigation to be flexible in quenchers’ output data processing) can be integrated in next designs at the expense of less than 0.1 mm^2^ chip area and a few mW of additional power consumption.

## Methods

The bit streams were obtained and analyzed as described in the following. The 4-channel LeCroy Waverunner 204Xi oscilloscope sampled the 4 quenchers’ output signals simultaneously each at 5 GS/s with a resolution of 8 bit. The received bit streams were stored in 10 blocks with a duration of 2 ms each by the oscilloscope (i.e. a 20 ms data stream at 50 Mbit/s corresponds to 1 million bits) and analyzed by comparing to the well-known PRBS-7 sequence in MATLAB on a personal computer. It should be mentioned that the length of the PRBS was not limited by the SPADs but by AC-coupling of the light source. The differences between sent and received bits were counted by MATLAB as errors and divided by the number of compared bits (i.e. by 10^6^, the number of bits sent) to determine the BER.

### Digital latch-type processing

**Figure 7:**
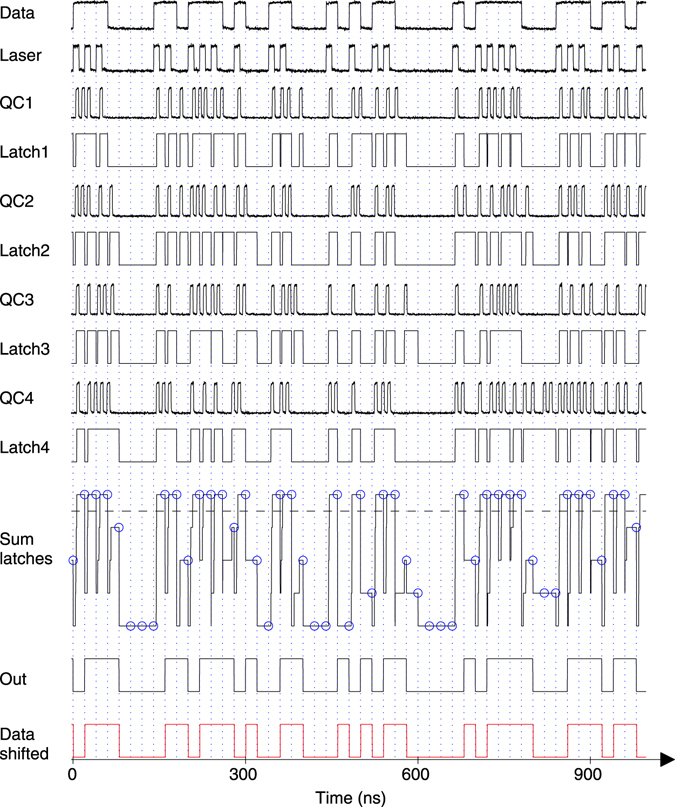
Digital latch-type processing of the 4 quencher circuits (QC) output data for 50 Mbit/s RZ at an optical power of 7.5 nW. The signals for a sequence of 50 bits were exported from Matlab. The PRBS7 “Data” signal was measured at the output of the bit pattern generator and read into Matlab. For the measurement of the “Laser” signal the duty cycle in the bit pattern generator was set to 50% to obtain RZ instead of NRZ. “Data” and “Laser” signals were measured once before the experiments (this was sufficient since PRBS7 repeats after 127 bits). The signals QC1, QC2, QC3 and QC4 are simultaneously measured QC output signals. The “Latch1” to “Latch4” curves are calculated by MATLAB. The “Sum latches” is calculated by MATLAB as the sum of the 4 latch signals. The dashed line represents the decision threshold used by MATLAB in order to determine at the end of each bit whether a “1” or “0” was detected. The blue circles represent the values taken for the decisions. The final output data “Out” are therefore shifted by one bit period to the right compared to the input data “Data”. For easy comparison, the input data “Data” are shifted by one bit to the right and are added as “Data shifted” at the bottom of the figure (red curve).

In MATLAB processing, a latch is assumed at each of the four buffer outputs of the test chip. As depicted in Fig. 7 the latch is set by the positive edge of the QC output, i.e. when the quencher detects an event. This state of the latch is kept until the new bit period starts. For the measurements a logical “1” is obtained only when all 4 latches were set during the corresponding bit period. BER was obtained in Matlab by comparing “Out” with “Data shifted”, counting the number of different bits as errors and dividing by the total number of bits.

### Analog processing

**Figure 8:**
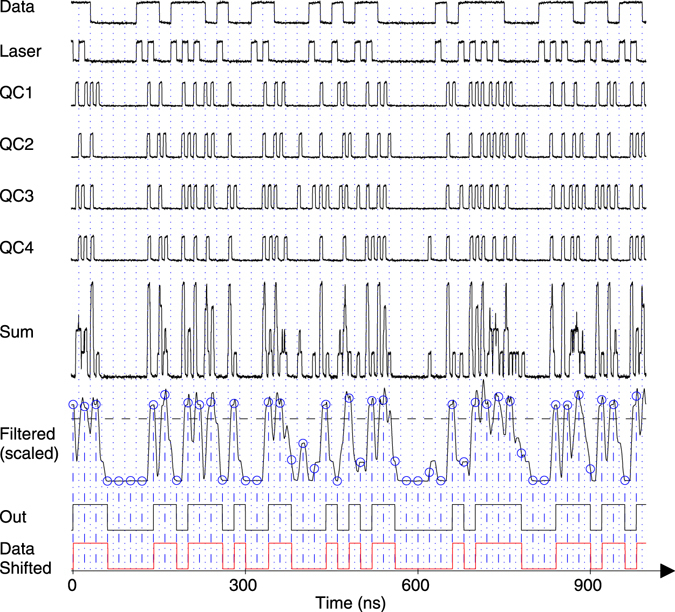
Analog processing of the 4 quencher circuits (QC) output data for 50 Mbit/s RZ at an optical power of 7.5 nW. The signals for a sequence of 50 bits were exported from Matlab. The signals Q1, Q2, Q3 and Q4 were measured simultaneously with the oscilloscope and imported into Matlab; their sum “Sum” was calculated by Matlab. The “Filtered” signal was scaled by a factor of 2 to make the curve progression better visible. The dashed line represents the threshold used by Matlab. The final output data (“Out”) are shifted by half a bit period to the right compared to the input data “Data” because the decision is made in the middle of each bit (denoted by the circles). The input “Data” sequence is shifted by half a bit (i.e. by 10 ns) to the right and repeated as “Data Shifted” to make a better comparison of output data “Out” and input “Data” possible.

For the analog processing approach with MATLAB the four QC output voltages are added, as depicted in Fig. 8. This sum is then filtered by a moving average filter to smoothen the signal. Finally, a decision threshold is used to obtain a logical “0” or “1” at defined sampling points.

The window lengths of the moving average filter were 61 (61 samples at 5 GS/s correspond to a length of 12 ns; the duration of a bit at 50 Mbit/s is 20 ns) for the return to zero 50 Mbit/s measurements, 91 (corresponding to 18 ns) for the non-return to zero 50 Mbit/s measurements, and 51 (corresponding to 10 ns; the duration of a bit at 100 Mbit/s is 10 ns) for the return to zero 100 Mbit/s measurements. In a hardware realization the moving average filter can be replaced by a simple low pass filter and the decider can be implemented by using a comparator. It should be possible to further optimize the analog processing approach by utilizing more advanced filter topologies. BER was obtained in Matlab by comparing “Out” with “Data shifted”, counting the number of different bits as errors and dividing by the total number of bits.

**Figure 9:**
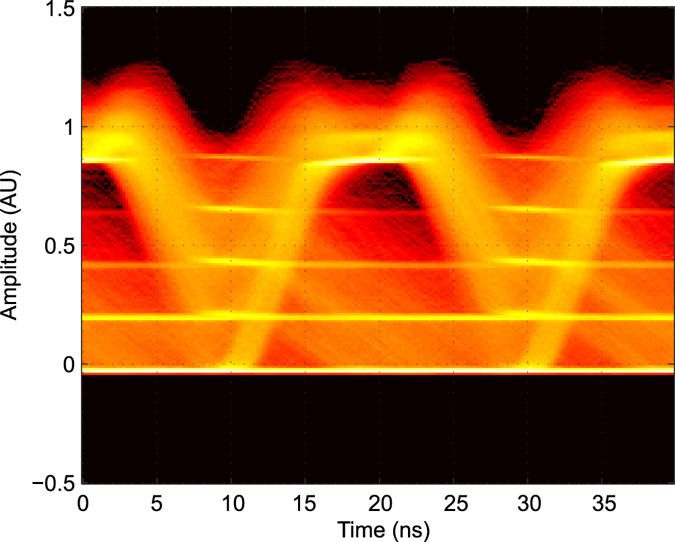
Eye-diagram for analog processing of the 4 quencher circuits (QC) output data for 50 Mbit/s RZ at an optical power of 7.5 nW. The filtered signal of the sum of the 4 latch outputs from Matlab was used to construct the eye-diagram. The clock from the bit pattern generator, which generated the Data signal was used as trigger to overlay 90.000 bits.

To show the symbol-dependent hysteresis the eye-diagram shown in Fig. 9 was constructed from the filtered sum latches signal obtained by Matlab. The moving average filter using a length of 12 ns was implemented in Matlab. Four (not counting the zero line as a fifth) bright horizontal lines are visible in Fig. 9, which are caused by the addition of the four quenchers’ digital output signals. Within an eye of about 0.15 AU (arbitrary unit) for a duration of about 5 ns (between about 18 ns and 23 ns), the density of traces is lowest between the third and fourth bright line. The decision threshold of a comparator has to be set to the middle between the third and fourth bright line. This eye is large enough for applying a clocked comparator successfully. The quality of the eye is limited by the BER of 4 × 10^−4^ and the analog processing method used. When considering the trace “Filtered (scaled)” in Fig. 8, the limited quality of the eye is not surprising. There is, however, room for further work to find a better method of analog processing of the quenchers’ output data.

**Figure 10:**
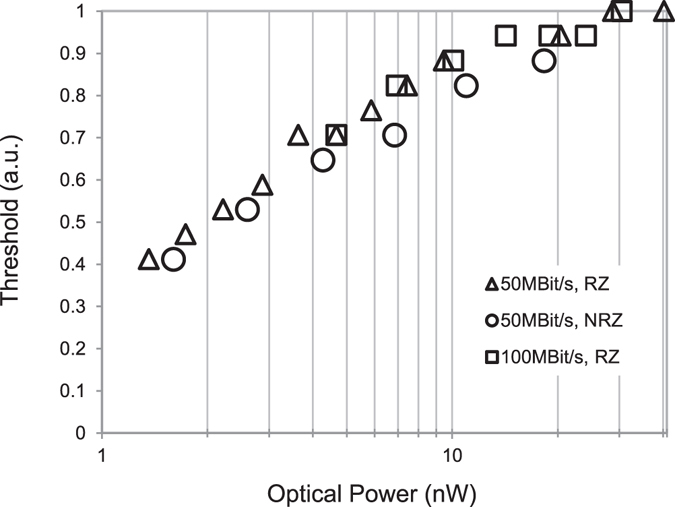
Optimized decision threshold over average optical input power for analog post-processing.

A better sensitivity was obtained with an optimized decision threshold for different optical input powers. Figure 10 shows the optimum threshold values used for processing of the quenchers’ output data. Such an adaptive decision threshold can be realized with a dedicated analog circuit or a digital counter plus a digital to analog converter setting its output voltage in an appropriate manner in dependence on the input optical power or count rate of the QCs, respectively.

## Electronic supplementary material

Supplementary Information
