# Supplementary material for: Integrated fiber optical receiver reducing the gap to the quantum limit
Source: Sci Rep. 2017 Jun 1;7:2652. doi: 10.1038/s41598-017-02870-2 (PMC5454008; doi:10.1038/s41598-017-02870-2)
Supplement: Supplementary file 1 — Supplementary Information [file 41598_2017_2870_MOESM1_ESM.pdf]

Supplementary information

Integrated fiber optical receiver reducing the gap to the quantum limit

Horst Zimmermann*, Bernhard Steindl, Michael Hofbauer, Reinhard Enne

I. Quantum limit and sensitivity

The quantum limit follows from the Poisson statistics

$$p_m(k) = \frac{m^k}{k!} e^{-m} \qquad (1)$$

where *m* is the average number of events and $p_m(k)$ is the probability that *k* events happen when in average *m* events occur.

For a receiver with one photodiode and a bit error rate (BER) of 10^{-9} (For such a low BER usually no error correction is necessary.) the probability that a "0" is detected has to be smaller than 10^{-9} when a logical "1" is sent, when the quantum efficiency of the detector is 100%. According to (1), 21 photons have to be sent in average for a "1". For a BER of less than 2×10^{-3} at least 7 photons are needed.

Due to the parasitic effects (dark count rate, afterpulsing probability 5.1%, optical crosstalk probability OCP 2.3%) of an SPAD a logical "1" can be detected although a logical "0" was sent. The most critical effect is, when in two SPADs an afterpulse occurs in the same bit period, leading to a BER of about 2.5×10^{-3}. Another critical event is when an SPAD shows an afterpulse and causes an optical crosstalk (APP-OCP: $5.1\times10^{-2} \times 2.3\times10^{-2}$) in a neighboring SPAD, leading to a probability of about 1.2×10^{-3}. Because there are dark counts in addition, a receiver consisting of two SPADs possessing the mentioned parasitic properties is not sufficient in order to be able to perform error correction, where a BER of less than 2×10^{-3} is necessary. A receiver with three SPADs would lead to a BER of larger than 1.3×10^{-4} (The worst combination of errors comes from an SPAD APP of 5.1%, when an afterpulse in each of the three SPADs occurs in the same bit, leading to a probability of $(5.1\times10^{-2})^3=1.3\times10^{-4}$. Because the error probabilities from other combinations like APP-APP-OCP, APP-OCP-OCP, DCR-APP-APP, DCR-APP-OCP and so on have to be added, we assumed that three

SPADs will not be sufficient also because of additional errors due to jitter for instance. The probability of APP[4], however, is 6.3×10^{-6}, leaving enough room to the BER limit of 2×10^{-3} also when considering the other combinations. Therefore we designed a receiver with four SPADs. The experiments indeed showed that four SPADs are necessary for a low enough BER. As a consequence a low-enough BER of a "0" will increase the average number of sent photons for a "1" by a factor equal to the number of necessary SPADs, i. e. by a factor of 4 for the receiver introduced here.

The average optical power P_{opt} corresponding to the number of photons can be calculated according to

$$P_{opt} = \frac{m\, h\nu\, B}{2} \qquad (2)$$

where $h\nu$ is the photon energy and B is the data rate in s^{-1}.

The sensitivity is defined as the average optical power which is necessary to achieve a certain BER. The sensitivity is often given in dBm ($10 \log(P_{opt}/1mW)$).

II. Thin SPAD versus thick SPAD, capacitance and avalanche charge

Many SPADs integrated in CMOS were reported, which have a thin combined absorption and multiplication region at a p+/n-well junction or at a p+/deep-n-well junction [S1,S2,S3,S4,S5]. Fig. S1 compares these thin SPADs with the thick SPAD investigated here.

The thickness of the space-charge region within the n-well or deep n-well in these thin SPADs (see right part of Fig. S1) is in the order of only 1µm. In 0.13µm CMOS, the thickness of the space-charge region was only 0.3µm. [S5] Charge carriers photogenerated in the p-substrate below the (deep) n-well do not reach the multiplication region (because the (deep) n-well is connected to a positive bias voltage) and cannot start an avalanche process. Therefore the photon detection probability of thin SPADs for red is rather low and their junction capacitance is rather large.

The thick SPAD (see left part of Fig. S1) possesses a space-charge region with a thickness of 12µm for absorption of more photons in the red spectral range. The electrons photogenerated in the thick

absorption zone drift upwards to the multiplication zone and can trigger the avalanche process there leading to a larger PDP for red and near-infrared light than of the thin SPADs. Charge carriers photogenerated in the substrate below the (deep) n-well of the thin SPAD do not reach the multiplication zone and do not trigger an avalanche.

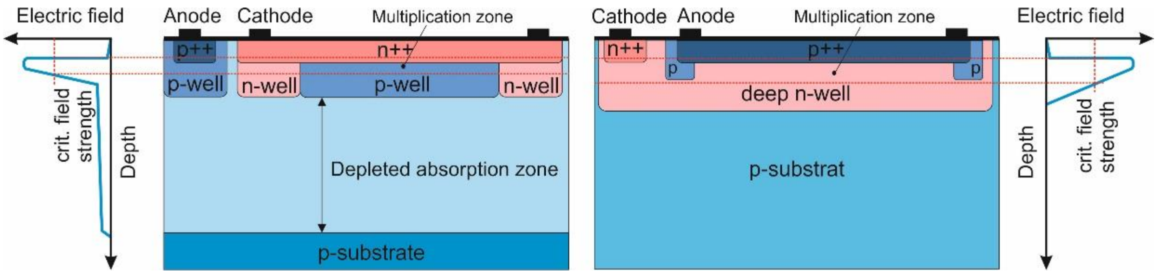

Fig. S1. Comparison of cross sections and electric field distributions of thick SPAD (left) and thin SPAD (right). The thick SPAD contains a thick absorption zone in addition to the multiplication zone; whereas the thin SPAD uses a thin combined multiplication/absorption zone.

Recent work [S6] implemented a thicker multiplication region, which however still was limited by the penetration depth of the deep n-well in 0.18µm CMOS technology. Nevertheless at 11V excess bias, an impressive PDP of 43% for 600nm, DCR of 1.5cps/µm² and an APP of 7.2% (for 300ns dead time) were reported. [S6] A thicker epitaxial structure was used for an SPAD with a PDP of 62.2% to 64.8% at 610nm and at an excess bias of 5V [S7,S8]. DCR of best SPADs was 0.6cps/µm² and mean DCR density was 7.6cps/µm² at an excess bias of 5V. APP was 1.63% for a dead time of 17.9ns at 5V excess bias. However, a proprietary customized CMOS process with special n- and p-type layers was used. The capacitance was not reported.

The SPAD suggested here possesses an about 12µm thick separate absorption zone and its capacitance C is about a factor of 10 lower as that of thin SPADs. Figure S2 shows the capacitance of the suggested thick SPAD measured with an Agilent 4284A Precision LCR Meter. The p-well depletes completely for voltages exceeding 16V and the thick absorption zone depletes fully at approximately 18V. The large step at about 17.5V in Fig. S2 also indicates that the thick SPAD

presented here has about a factor of 10 lower capacitance than thin SPADs formed by p+/(deep)n-well junctions.

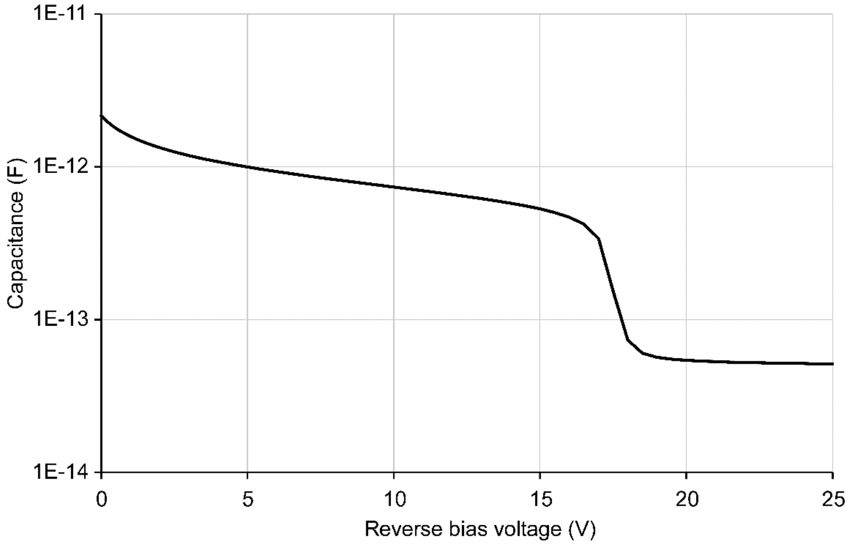

Fig. S2. Capacitance over voltage of an SPAD with the cross-section shown in Fig. 2 and having an area of 3,750 µm².

Since the afterpulsing probability increases with the amount of charge carriers being generated in the avalanche process, because more traps can be filled by more available charge carriers, a smaller capacitance of the SPAD reduces the amount of charges, which can be captured by traps. With an active quencher the SPAD's cathode is left floating after it has been charged to V_{plus}. The avalanche current discharges the SPAD's capacitance C and the maximum available charge is C times V_{plus}. A fast quencher, however, discharges the SPAD's capacitance via the quencher and reduces therefore the amount of charge carriers within the avalanche. It can be expected that the thick SPAD shows a much lower avalanche charge Q and afterpulsing probability than thin p+/(deep-)n-well SPADs.

$$Q = C\, U_{dth} \qquad (3)$$

A low detection threshold U_{dth} (100mV for the comparator used) of a very fast quenching circuit in addition helps to keep the avalanche charge flowing through the SPAD low. For the quencher used, passive and active quenching cause a larger avalanche charge and a value up to the excess bias

voltage of the SPAD has to be inserted into Eq. (3) instead of V_{dth}. The exact voltage value depends on the time needed for quenching. The capacitance is reduced by about a factor of 10 by the thick p- epi layer (like in a pin photodiode), compared to thin SPADs, which results in a clear reduction of the quenching/avalanche charge.

III. Isolation of circuits

The avalanche photodiode needs a much larger bias voltage than the circuit supply voltage. The anode of the SPAD is formed by the substrate of the complete chip and therefore has to be biased with a negative voltage compared to the circuit supply voltage. Circuits on the same chip together with the SPAD are only possible when the transistors can be isolated from this negative substrate voltage. The process used is a so-called triple-well CMOS process, which offers a deep n-well as a third well. Fig. S3 shows a cross section of the SPAD together with transistors to explain the isolation of the circuits. The deep n-well is connected to the most positive potential in the circuit, i. e. to V_{plus}. In such a way the junction formed by deep n-well and p-substrate is always in reverse direction.

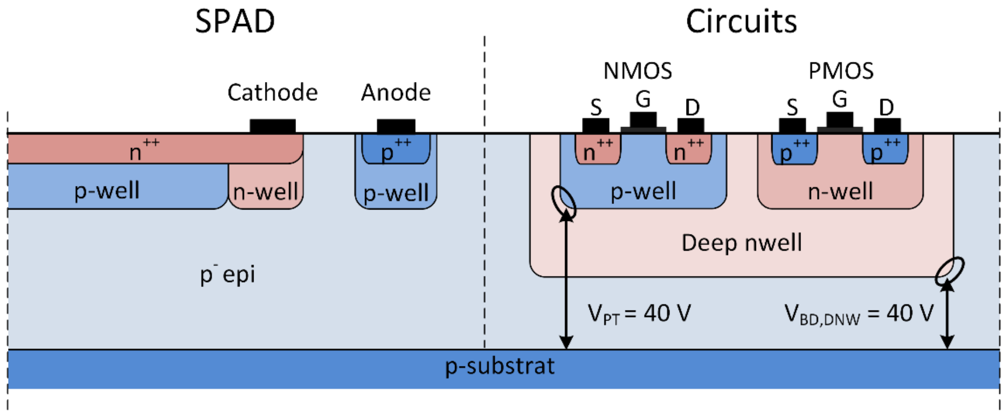

Fig. S3. Principle of isolating the transistors from the strongly negative anode voltage of the SPAD.

The deep n-well possesses a punch-through voltage V_{PT} of about 40V towards the substrate (see Fig. S3), i. e. the n-channel MOSFETs can be isolated inside a deep n-well from the negative anode

of the SPAD. The deep n-well is also used to increase the breakdown voltage of the n-well, in which the p-channel MOSFETs are located, towards substrate. The breakdown voltage of deep n-well towards the substrate $V_{BD,DNW}$ is larger than 40V (see Fig. S3) in the CMOS process used. This breakdown occurs at the edge as marked in Fig. S3. In such a way, circuits can be operated together with the thick SPAD on the same chip. These high breakdown voltages represent a clear advantage of the 0.35µm pin-photodiode CMOS process used over nanometer CMOS technologies with very limited breakdown voltages.

IV. Spectral dependence of the photon detection probability

The spectral dependence of the PDP was measured by illuminating the SPAD with a constant photon flux. This photon flux was generated by means of a tunable light source built by the Xenon light source ASB-XE-175 and the monochromator CM110, both from Spectral Products, as well as by a fiber coupled tunable optical attenuator DD-100-11 from OZOptics. The photon flux for each wavelength was calibrated by a standard Si PIN photodiode (S5971). This photodiode was cooled down to a temperature of -30°C in order to reduce its leakage current down to the femtoampere range. The PDP was then obtained by stepping the wavelength while illuminating the SPAD and by building the ratio between the pulse rate at the output of the quencher (corrected for afterpulses and dark counts) and the photon flux.

The spectral dependence of the photon detection probability (PDP) of the SPAD with the cross section of Fig. 2 for two quenching voltages V_q (breakdown voltage V_{bd}=25.8V) is shown in Fig. S4. There is a ripple due to optical interference in the isolation and passivation stack of the standard 0.35µm CMOS process. In Fig. S4 additionally the PDPs measured with the 635nm laser used for the bit error measurements are indicated by markers. It can be concluded however from Fig. S4 that the PDP is largest in the red spectral range. At 850nm and for V_q=6.6V the PDP of 25% is more than three times larger than that of the thin SPAD described in ref. 10.

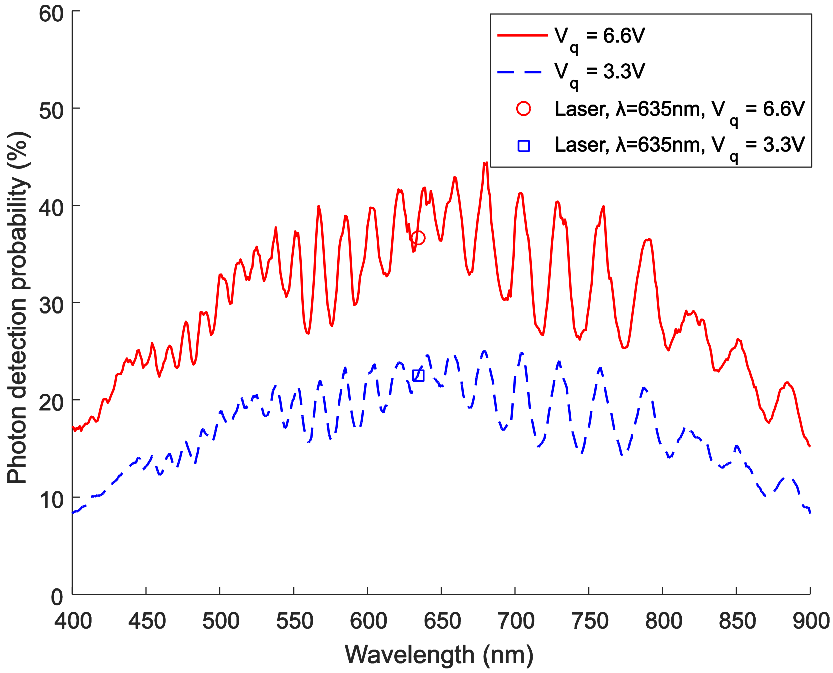

Fig. S4. Measured photon detection probability (PDP) of a SPAD reference device.

V. Dark count rate

The dark count rate at 25°C was measured at a reference SPAD having the same shape and an area of the multiplication zone of 3,750µm² (as depicted in Fig. 2) in dependence on the excess bias voltage. Fig. S5 shows the obtained results. At an excess bias voltage of 3.3V the DCR is 21.5kcps (5.7cps/µm²) and at 6.6V excess bias the DCR is 35.5kcps (9.5cps/µm²).

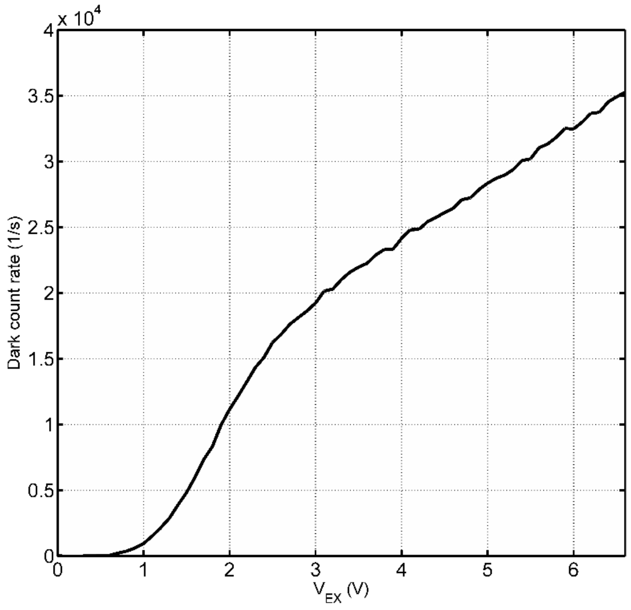

Fig. S5 Measured dark count rate of a reference SPAD with 3,750µm² active area.

VI. Measurement set-up

Figure S6 depicts the measurement set-up used for the characterization of the SPAD-based receiver. The light source was stabilized at constant temperature and with a control loop using a monitor photodiode. The optical receiver was mounted on a Peltier element and kept at 25°C by a thermoelectric cooler (TEC) controller. The set-up was controlled by a PC with a Labview measurement program. After calibration the fiber was moved from the optical power meter to the SPAD receiver.

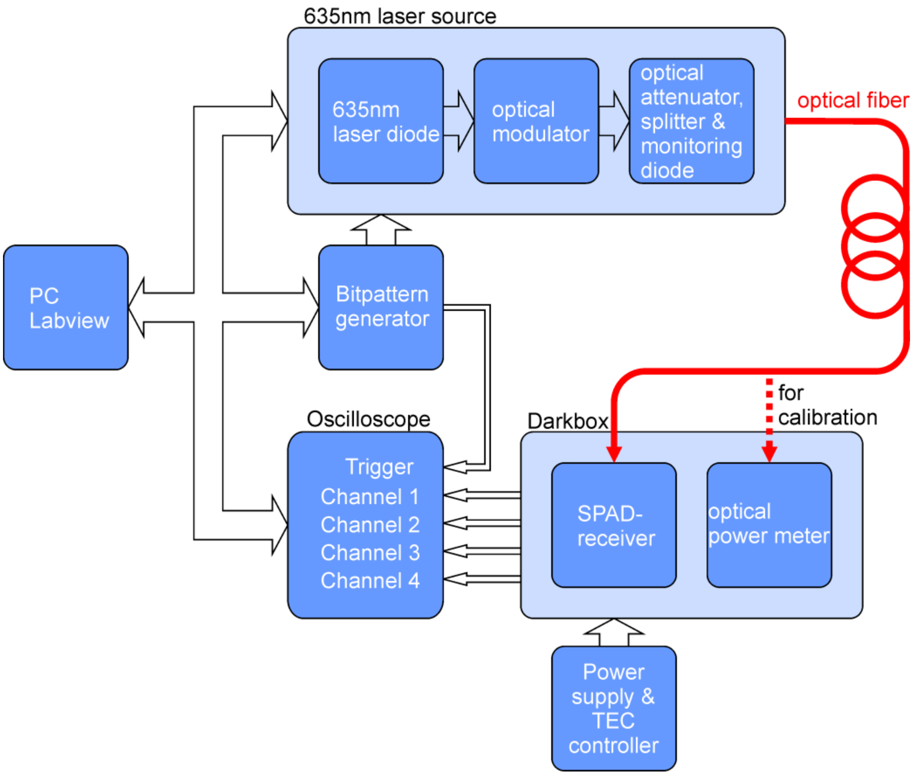

Fig. S6 Block diagram of the measurement set-up

S1. Finkelstein, H., Hsu, M. J., Esener, S. C., STI-bounded single-photon avalanche diode in a deep-submicrometer CMOS technology, IEEE Electron Device Letters **27**, 887-889 (2006).

S2. Richardson, J. A., Grant, L. A., Henderson, R. K., Low dark count single-photon avalanche diode structure compatible with standard nanometer scale CMOS technology, IEEE Photon. Technol. Lett. **21**, 1020-1022 (2009).

S3. Niclass, C. et al., Design and characterization of a 256×64-pixel single-photon imager in CMOS for a MEMS-based laser scanning time-of-flight sensor', Opt. Exp. **20**, 11863-11881 (2012).

S4. Maruyama, Y., Blacksberg, J., Charbon, E., A 1024×8 700 ps time-gated SPAD line sensor for laser Raman spectroscopy and LIBS in space and rover-based planetary exploration', Paper presented at *IEEE ISSCC* 2013, San Francisco, 110-111.

S5 Ray, S., Hella, M. M., Hossain, M. M., Zarkesh-Ha, P., and Hayat, M. M., "Speed optimized large area avalanche photodetector in standard CMOS technology for visible light communication," Proceedings IEEE SENSORS 2014, Valencia, 2014, pp. 2147-2150.

S6. Veerappan, C. and Charbon, E., A Low Dark Count p-i-n Diode Based SPAD in CMOS Technology," IEEE Transactions on Electron Devices **63**, 65-71 (2016).

S7. Niclass, C. et al., T., A NIR-Sensitivity-Enhanced Single-Photon Avalanche Diode in 0.18µm CMOS. Paper presented at *Int. Image Sensor Workshop* 2015, paper 11-4.

S8. Takai, I. et al., Single-Photon Avalanche Diode with Enhanced NIR-Sensitivity for Automotive LIDAR Systems, Sensors **16**, 459 (2016).
